# Quality control of mRNAs at the entry of the nuclear pore: Cooperation in a complex molecular system

**DOI:** 10.1080/19491034.2018.1439304

**Published:** 2018-03-14

**Authors:** Mohammad Soheilypour, Mohammad R. K. Mofrad

**Affiliations:** Molecular Cell Biomechanics Laboratory, Departments of Bioengineering and Mechanical Engineering, University of California, Berkeley

**Keywords:** Mechanotransduction; mRNA export; nucleocytoplasmic transport; nuclear pore; gene regulation

## Abstract

Despite extensive research on how mRNAs are quality controlled prior to export into the cytoplasm, the exact underlying mechanisms are still under debate. Specifically, it is unclear how quality control proteins at the entry of the nuclear pore complex (NPC) distinguish normal and aberrant mRNAs. While some of the involved components are suggested to act as switches and recruit different factors to normal versus aberrant mRNAs, some experimental and computational evidence suggests that the combined effect of the regulated stochastic interactions between the involved components could potentially achieve an efficient quality control of mRNAs. In this review, we present a state-of-the-art portrait of the mRNA quality control research and discuss the current hypotheses proposed for dynamics of the cooperation between the involved components and how it leads to their shared goal: mRNA quality control prior to export into the cytoplasm.

## Introduction

**Figure 1.**
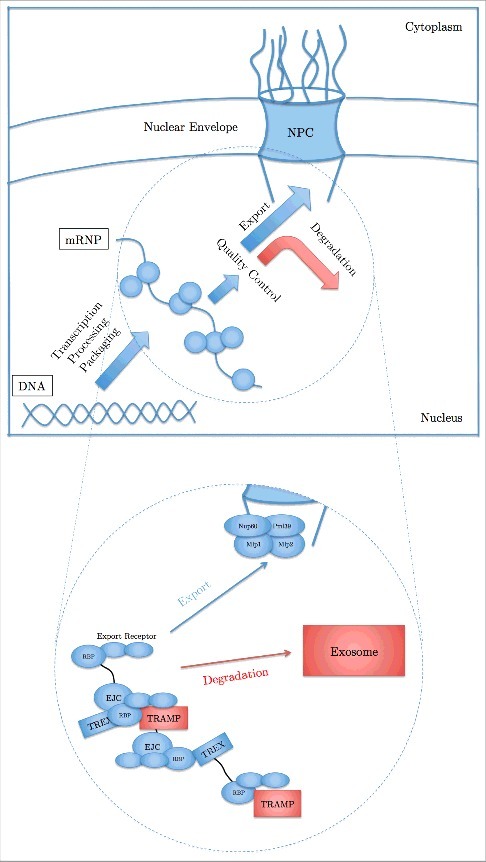
mRNA biogenesis in eukaryotic cells. Upon transcription, mRNA undergoes some processing and packaging steps, leading to the formation of messenger ribonucleoprotein (mRNP). Prior to export, mRNPs are quality controlled and either exported through the nuclear pore complex (NPC) into the cytoplasm or retained and degraded inside the nucleus. Successfully exported mRNPs engage in the translation process to produce proteins. Insert: The two pathways of mRNA's fate after transcription inside the nucleus. The export pathway (blue) involves multiple proteins and complexes including Exon junction complex (EJC), RNA-binding proteins (RBPs) (e.g. Nab2, Npl3, Gbp2, and Hrb1 in yeast [10, 12, 13] and 9G8, SRp20, and ASF/SF2 in vertebrates [14]), and the TREX complex. Once mRNA undergoes all the processing and packaging steps, export receptor heterodimers are recruited to facilitate the export of the mRNP complex. On the other hand, in the case of aberrant mRNAs (red), e.g. unspliced transcripts, RBPs recruit the TRAMP complex, which facilitates the degradation of mRNA by the nuclear exosome. Nuclear pore complex (NPC) associated quality control proteins (primarily Mlp proteins) ensure that only normal mRNAs are passing through the NPC and aberrant mRNAs are retained inside the nucleus for subsequent degradation by the nuclear exosome.

Transport of messenger ribonucleic acids (mRNAs) from the nucleus into the cytoplasm is fundamental to various cellular functions in eukaryotes. Mutations or lacking of the components in mRNA export machinery have been linked to different human diseases [1,2]. mRNAs are exported through the nuclear pore complexes (NPCs), the nanochannels that perforate the nuclear envelope (NE) and primarily act as a gateway for transport of various types of cargos (including mRNAs) into and out of the nucleus (see [3–8] for recent reviews on different aspects of NPC structure and function). Upon transcription inside the nucleus and prior to being exported into the cytoplasm, mRNAs are quality controlled to ensure the production of appropriately functioning proteins in the cytoplasm (Figure 1) [9]. However, the mechanisms by which aberrant mRNAs, e.g. unspliced, are recognized and retained inside the nucleus are poorly understood [10,11]. In this review we present recent findings on mRNA quality control mechanisms, specifically at the entry of the nuclear pore complex (NPC), and the two hypotheses on the underlying dynamics of these processes. While one hypothesis highlights the “switch-like” behavior of the involved proteins as the key for mRNA quality control, an alternative hypothesis suggests that the efficient quality control is the emergent behavior of a combination of different regulated stochastic interactions between the involved components.

## Export of mRNA transcripts from the nucleus into the cytoplasm

Before discussing mRNA quality control inside the nucleus, we will briefly present the main components of mRNA export system (for more comprehensive reviews on mRNA export see [15–17]). The processing and packaging steps prepare a complex of mRNA and various proteins and protein complexes, collectively called messenger ribonucleoprotein (mRNP), enabled to exit the nucleus through the NPC and engage in production of proteins in the cytoplasm (Figure 1). The NPC is filled with a set of intrinsically disordered proteins called FG (phenylalanine-glycine) nucleoporins or FG Nups that form a barrier for transport of cargos. Nuclear transport is, therefore, limited to either small molecules (∼20-40 kDa, diameter ∼5-9 nm) that could freely diffuse through this barrier or macromolecules (> 40kDa up to ∼25 MDa, diameter of up to ∼40 nm) that are bound to a specific set of proteins, called transporters or karyopherins. Transporters interact with FG Nups via their hydrophobic patches and carry the cargo through the nuclear pore [18]. In the case of mRNA export, transporters are called nuclear transport receptors (NTRs) or export receptors, which enable the mRNA to pass through the NPC. However, mRNAs do not directly recruit export receptors. Instead, RNA-binding proteins (RBPs) are key mediators that, on one end, bind to mRNA while, on the other end, recruit export receptors (namely, NXF1/NXT1 or Tap/p15 or Mex67/Mtr2), enabling the mRNA to interact with FG Nups and pass through the NPC. To date, several different RBPs such as Npl3 (associates with mRNA close to the 5’ cap) [12], Nab2 (associates with mRNA at the 3’ end) [13], Gbp2 and Hrb1 (associate with mRNA during splicing) [10] in yeast, and 9G8, SRp20, and ASF/SF2 in vertebrates [14], have been identified to facilitate acquisition of export receptors to mRNAs.

Although RBPs are considered as the main mediators in recruitment of export receptors, this process may involve other participating factors. The exon junction complex (EJC), deposited 24 nucleotides upstream of exon-exon junctions upon splicing, is suggested to mediate the recruitment of export factor (NXF1) to mRNAs [19,20]. However, analysis of human EJC and RNA interactomes reveals a physical association between EJC and SR proteins, which are RBPs featuring long repeats of serine and arginine amino acid residues. This observation might be a potential explanation for the functional overlap between EJC and RBPs [20]. In addition, both Yra1 and its metazoan homologue Aly/REF interact directly with export receptors [13,21–23]. However, Aly/REF is found not to be essential for mRNA export in *Drosophila* or *Caenorhabditis elegans* [23,24] and Yra1 is shown to be dispensable for mRNA export when an RBP (Nab2) and the export receptor (Mex67) in yeast are overexpressed. Therefore, Yra1 and Aly/REF are suggested to act more as cofactors for stabilization of the interaction between some of the RBPs and the export receptor [13]. It is worth noting that, on the other hand, some studies have identified Aly/REF as a required factor for efficient mRNA export [25,26]. Interestingly, it is also suggested that some genes can tether to NPC components, which regulates mRNA expression [27].

Although export of mRNA transcripts through the NPC is widely studied to date, the dynamics of mRNA export is still elusive. One of the main challenges is the lack of experimental methods that could capture the dynamics of mRNA export with a high spatial and temporal resolution. Experimental approaches such as oligo(dT) *in situ* hybridization assay or single molecule fluorescence *in situ* hybridization (smFISH) can primarily perform bulk measurements to determine the interacellular distribution of RNA but cannot capture high-resolution *in vivo* dynamics [15]. Recent advancements in RNA labeling as well as imaging methods, however, have provided a platform to capture spatial and temporal dynamics of individual mRNAs *in vivo* [28–31], which enables researchers to explore mRNA export dynamics with a higher resolution both in time and in space. Moreover, recently developed computational models of mRNA export provide high-resolution (nanometer and microsecond) details of mRNA export in long time scales (seconds) [32], enabling researchers to evaluate the role of different factors and assess the effect of different parameters, e.g. affinities or expression levels.

## Quality control of mRNAs is a complex system involving a multitude of cooperating factors

To date, various methods and approaches have been employed to identify the underlying mechanisms of mRNA quality control. Using an array of techniques mostly involving knock out/knock down and/or mutation of target proteins, several proteins and protein complexes have been implicated in this process (for example see [10,33–36]). Some of these components are proteins/protein complexes that bind to mRNA, e.g. RBPs, as adapters that facilitate various stages of mRNA biogenesis. Other involved factors interact with these mRNA-bound components to fulfill these processes. While current research has identified various pieces of mRNA quality control machinery by identifying the different cellular components involved, details of the underlying mechanism are still unclear. Here, we have summarized the major factors involved in mRNA export, quality control, and nuclear degradation in Table 1. In the next section, more details are provided regarding the role of each of these factors in their respected processes.

**Table 1.** Proteins and protein complexes involved in mRNA export, quality control, and degradation. Yeast factors are presented with their metazoan counterparts in parentheses.

Protein or protein complex	Reference(s)
Mlp1 (Tpr)	[10, 33, 34, 36, 37]
Mlp2 (Tpr)	[38-40]
Nab2 (ZC3H14)	[41-45]
Npl3	[12, 45, 46]
Gbp2 and Hrb1	[10, 45, 47]
Pml39	[35]
TRAMP complex (NEXT complex)	[10, 11, 48, 49]
Nuclear exosome	[50]
TREX complex	[10, 51, 52]
Yra1 (Aly/REF)	[13, 21, 22]
Mex67/Mtr2 (Tap/p15 – NXF1/NXT1)	
Exon junction complex (EJC)	[19, 20]

## NPC proteins inhibit export of aberrant mRNAs

Under normal conditions, aberrant mRNAs that reach the NPC are not allowed to pass through, instead they are retained inside the nucleus and subsequently degraded. The NPC quality control step is achieved by a set of nuclear pore associated proteins including Mlp1, Mlp2, Pml39, and Nup60 [10,33–37,53]. Among these, Mlp1 and Mlp2 (homologues of their human counterpart Tpr) are the most studied proteins and appear to be the main role players in NPC-associated quality control [10,34,36,37,54,55]. Pml39 and Nup60 are suggested to be upstream effectors for Mlp1 to localize it to the nucleoplasmic side of the NPC [33,35,56]. While Mlp1 and Mlp2 are shown to associate with mRNPs [38,39,54,57] they have no essential role in mRNA export [40,57,58]. However, overexpression of Mlp1 leads to mRNA accumulation in the nucleus and its deletion results in pre-mRNA leakage [33,59]. Mlp2 is also suggested to function in quality control based on its enhanced interaction with mRNPs assembled in Yra1 mutant cells [57]. The interaction of Mlp proteins with a multitude of mRNP components suggests that they function as a checkpoint for maturity of mRNPs prior to their export through the NPC [10,37,38,54,57], allowing normally processed and packaged mRNAs to pass while retaining aberrant ones inside the nucleus. Interestingly, according to *in vivo* imaging studies, mRNAs spend 4–16 times more time at the nuclear basket compared with the central channel, which, besides mRNA remodeling at the nuclear basket, could be attributed to the quality control process [60,61]. However, it is worth noting that it has been recently shown that under stress, heat-shock mRNAs bypass the NPC-associated quality control step and are rapidly exported [62].

Retained aberrant mRNAs are marked by the yeast Trf-Air-Mtr4 polyadenylation complex (TRAMP) for degradation [10,11,48]. Some SR proteins are suggested to facilitate this process by enabling a proper recruitment of the TRAMP complex or stabilizing its binding to aberrant mRNAs [10]. The marked mRNAs will be subsequently degraded by the nuclear exosome, a multisubunit complex involved in processing and degradation of different types of RNAs [50]. Similar complexes and pathways are identified in human. The trimeric nuclear exosome targeting (NEXT) complex is required for exosomal degradation of promoter upstream transcripts (PROMPTs) [49].

Upstream of these steps, transcription and mRNA export are tightly coupled via the evolutionary conserved transcription/export (TREX) complex [51,52]. In yeast, this complex is composed of THO sub-complex (Hpr1, Tho2, Thp2, Mft1) and mRNA export adapter proteins (Sub2 and Yra1) [51]. Similarly, human counterpart of the THO complex as well as Aly and UAP56 constitute the human TREX complex [63]. TREX-2 complex is recently shown to stably associate with the nuclear basket [64]. However, despite the role of TREX complex in mRNA export, deletion of one of its elements (Mft1) as well as mutation of another element (Yra1) have no effect on the leakage of unspliced transcripts, implying that TREX has no direct role in the quality control of mRNAs [10].

Therefore, based on the findings to date, mRNAs are decorated with RBPs and once they undergo the required processing and packaging steps, RBPs recruit export receptors and facilitate the export of the resulting mRNP. On the other hand, Mlp proteins, at the nuclear basket, inhibit the export of aberrant mRNAs and RBPs that are bound to these mRNAs stabilize the binding of the TRAMP complex to facilitate their degradation (Figure 1). As a simplified analogy, mRNA could be considered as an individual attempting to attend an event by purchasing tickets (RBPs), where multiple tickets are required for attendance. Tickets (RBPs) need to be certified by export receptors to be accepted. Finally, Mlp proteins represent guards at the entry that check the tickets and only allow individuals with a minimum number of certified tickets to pass. Nonetheless, how RBPs manage to determine mRNA's fate and what the distinctive feature is that enables the cell to distinguish normal and aberrant mRNAs and retain the aberrant ones is under debate.

## Cooperation in a complex molecular system: How aberrant mRNAs are recognized by NPC components and retained inside the nucleus

**Figure 2.**
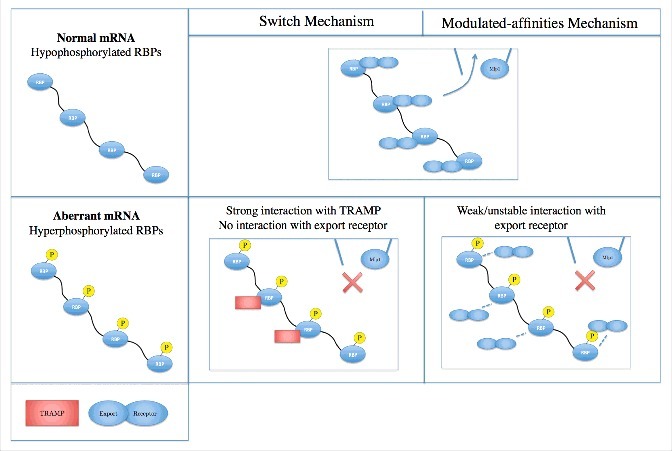
Comparison of mRNA quality control mechanism between the two hypotheses reviewed herein. The switch mechanism suggests that some RBPs, e.g. Gbp2 and Hrb1, do not interact with export receptors when bound to aberrant mRNAs, which is potentially achieved according to phosphorylation or methylation state of RBPs. Instead, these RBPs bind to the TRAMP complex for mRNA degradation. On the other hand, in the modulated-affinities mechanism, aberrant-mRNA-bound RBPs can still recruit export receptors, but with a low affinity. This hypothesis suggests that the weak/unstable interaction between hyperphosphorylated RBPs with export receptors is sufficient for the nuclear basket proteins, e.g. Mlp1, to distinguish aberrant mRNAs and retain them inside the nucleus (please refer to the text for more details).

Various hypotheses are proposed regarding how mRNAs are quality controlled inside the nucleus [65]. For example, an interesting mRNA biogenesis model suggests that mRNA quality control is a result of kinetic competition between mRNA processing and degradation, which is thoroughly discussed before [66]. Here, however, we primarily discuss the hypotheses that consider the NPC components as essential parts of mRNA quality control. Quality control of mRNAs at the entry of the NPC is achieved by cooperation between several different sets of proteins and protein complexes and various research groups have sought to unveil how these different components cooperate with each other. Hackmann et al. recently identified two SR proteins in yeast, namely Gbp2 and Hrb1, and suggested that they function as *switches* that according to the state of mRNA, i.e. processed or not, recruit export receptors or the TRAMP complex for export or degradation, respectively [10]. This mechanism identifies the SR proteins (which comprise most of the RBPs) as the key components to distinguish normal and aberrant mRNAs. This switch behavior is suggested to be achieved either according to the phosphorylation or methylation state of the SR proteins, or extended association of TRAMP. The latter suggests that mRNAs are initially associated with TRAMP and upon successful splicing, lose their association. Subsequently, SR proteins associate with export receptors, excluding their potential to bind to TRAMP [10] (Figure 2). Therefore, these SR proteins either bind to *export-promoting factors* or *degradation-promoting components*, therefore called switches [10] (we will call this mechanism as the *switch mechanism*).

Huang et al. previously studied two other SR proteins, namely 9G8 and ASF/SF2 in metazoans, and suggested that although the interaction of SR proteins with export receptors depends on whether mRNA is correctly processed or not, it only alters the affinity of the interaction, rather than completely eliminating the interaction; meaning that normal-mRNA-bound and aberrant-mRNA-bound SR proteins can both interact with export receptors. The results suggest that interactions between SR proteins and export receptors are modulated according to the state of mRNA, e.g. spliced or not [67]. SR proteins are hyperphosphorylated when they co-transcriptionally bind to pre-mRNAs, and, upon splicing, become hypophosphorylated, i.e. partially dephosphorylated [68]. Therefore, the phosphorylation state of SR proteins regulates their interactions with the target proteins. These SR proteins have been shown to be able to bind to export receptors when they are hyperphosphorylated (i.e. bound to pre-mRNAs), but with a lower affinity compared to when they are hypophosphorylated [67]. Therefore, SR proteins that are bound to aberrant mRNAs, and are hyperphosphorylated, could still recruit export receptors rather than behaving as a deterministic switch according to the state of mRNA (we will call this mechanism *modulated-affinities mechanism*) (Figure 2). It is worth noting that the switch mechanism and the modulated-affinities mechanism are not mutually exclusive. The former suggests that SR proteins either bind to export receptors or the TRAMP complex; however, it does not exclude the possibility of binding of aberrant-mRNA-bound SR proteins to export receptors with a lower affinity, which is suggested by the modulated-affinities mechanism.

Explaining the underlying mechanism of mRNA quality control using the switch mechanism is straightforward, where the ability to discern normal and aberrant mRNAs is attributed to the switch-like SR proteins, where aberrant mRNAs cannot recruit export receptors and, hence, are not able to interact with NPC proteins for export. However, it is not trivial to predict whether the modulated-affinities mechanism is sufficient for an efficient quality control of mRNAs, because in this hypothesis, aberrant mRNAs can still recruit export receptors and potentially get exported. From a complex systems standpoint, however, it is conceivable to hypothesize that the emergent behavior of the system, i.e. recognition and retention of aberrant mRNAs, is a result of the inter-molecular dynamics of the involved proteins with modulated affinities. This hypothesis, however, is not easily tractable using experimental approaches; partly due to the challenges in experimental studies that prevent researchers from exploring the *in vivo* dynamics of these processes and the factors involved with high spatiotemporal resolution [15]. Therefore, we recently developed a computational model of mRNA export and quality control using a complex systems approach, called agent-based modeling (ABM) [32,69]. We sought to identify the ‘minimal’ factors required for mRNA quality control, since it is still unclear which factors are necessary for a successful quality control. Accordingly, we developed a minimal model for mRNA quality control composed of RBPs, export receptors, and NPC-associated quality control protein (Tpr or Mlp1). Using the model, we evaluated whether only regulating the interaction between RBPs and export receptors is sufficient for nuclear basket quality control proteins to distinguish normal and aberrant mRNAs. Our results showed that a lower affinity of aberrant-mRNA-bound RBPs to export receptors could enable Tpr/Mlp1 to distinctively retain aberrant mRNAs (by binding to individual RBPs), while allowing normal mRNAs to pass through the NPC, implying that even without switch-like behavior of some SR proteins, mRNAs could be discriminated in this minimal system. Retention of aberrant mRNAs at the nuclear basket provides extra time for nuclear machineries to degrade mRNA or perform processing steps, e.g. splicing [70]. Our computational results imply that mRNA quality control does not necessarily require deterministic switches and, instead, the combination of regulated interactions could potentially discriminate normal and aberrant mRNAs (more on advantages of computational models in mRNA export and quality control in Box 1). It should be noted, however, that “*active”* involvement of the NPC and its constituents in mRNA export and quality control is still a matter of debate (please see further discussion in the “*Conclusion and prospects*” section).

**Figure 3.**
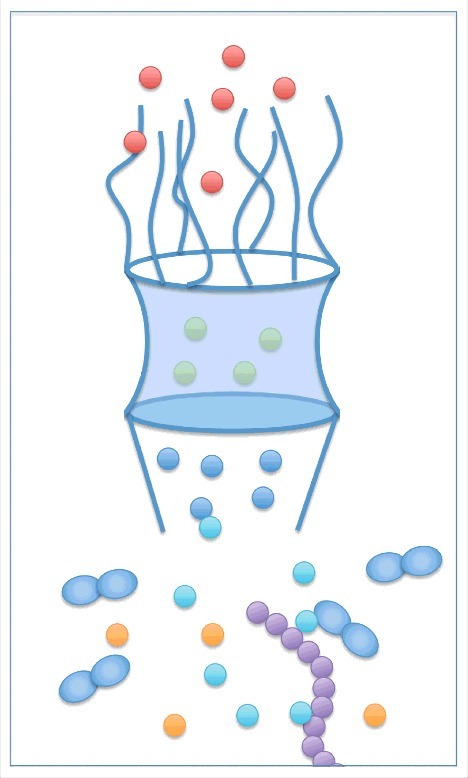
A schematic of agent-based modeling (ABM) of mRNA export and quality control. ABM is a bottom-up computational approach that simulates a complex system from the perspective of its constituents, molecules in this case. Here, each molecule is represented by a single agent, e.g. RBPs, or a multitude of agents, mRNA. Agents move and interact (bind and unbind) with other agents according to a set of pre-defined rules associated with biophysical properties of represented molecules (73, 74). Coarse-grained representation of molecules enables ABM to easily achieve high temporal scales while maintaining a relatively high spatial resolution. Therefore, ABM is uniquely suited to explore different aspects of mRNA biogenesis.

## Box 1. Benefits of computational models for mRNA export and quality control

Although mRNA export and quality control are explored with a range of experimental techniques, several unknowns still exist that are not easily tractable via experiments. For instance, the required density of export receptors that mRNA needs for an efficient export as well as how export receptor coverage on mRNA transcript affects mRNA export are still unknown [32]. In addition, the rate-limiting step of mRNA export through the NPC is still a matter of debate, with some experiments suggesting the nuclear basket [28,30], while others identifying the central channel of the NPC [71] as the rate-limiting step. Similarly, many aspects of mRNA quality control are still unclear. Besides the fact that the exact underlying mechanism is still a matter of debate (reviewed herein), the minimum required factors are also unknown. In addition, how mRNA length affects the quality control process is unclear.

Computational models enhance our understanding of biological systems by allowing us to explore hypotheses and evaluate the effect of different parameters on the system behavior. They could also lead to predictions that could explain an experimental observation or be further examined using *in vitro* or *in vivo* experiments. Accordingly, our group recently developed an agent-based model (ABM) to explore mRNA export and quality control (Figure 3) [32,69]. The model predicted that coverage of mRNA by export receptors affects export efficiency, with at least coverage of one mRNA terminus being necessary for a successful export. Furthermore, the nuclear basket was identified as the rete-limiting step in mRNA export, which is potentially associated with mRNA reconfiguring itself to thread into the central channel of the NPC. This observation could be further validated with quantitative single molecular imaging (SMI) of RNA molecules, which provides a higher spatial and temporal resolution of mRNA export [15]. In addition, we identified the minimum factors that ensure a successful mRNA quality control (detailed in the text). We predicted that it would be more challenging to identify and retain shorter mRNAs. Based on our simulations, longer mRNAs spend more time in the nuclear basket to form a compact conformation to initiate their export and, therefore, nuclear basket proteins have more time capturing and retaining them inside the nucleus. This computational prediction might be the reason that short mRNAs with fewer introns leak to the cytoplasm after spliceostatin A (SSA) treatment [65,72].

Computational modeling of mRNA export and quality control could be further employed to address other aspects of these processes as well. For instance, how distribution of RBPs on mRNA transcript affects the quality control process is not explored. The effect of other nuclear machineries, e.g. degradation, on mRNA quality control process is still under investigation. It is also still unclear whether the quality control process only selects normal mRNAs for export (selection model) or, instead, retains aberrant mRNAs inside the nucleus (retention model) [70] (please see *Conclusions and prospects* for more details).

## Conclusions and prospects

The switch mechanism and the modulated-affinities mechanism share the same core idea, i.e. SR proteins bind to different factors depending on their modulation state. However, the two mechanisms lead to two different perspectives of the mRNA quality control mechanism, which influences future directions for experiments. The former suggests that we should identify proteins that act as switches, while the latter suggests that we should study the dynamics of the system as a whole with higher spatial and temporal resolutions. Conventional experiments only allow for bulk measurements. Therefore, in most of the experiments, a potential component of the system is disturbed (e.g. knocked down) and the resulting effect, e.g. concentration of pre-mRNAs in the nucleus or the cytoplasm, is evaluated. These approaches only observe the system at discrete time intervals, ignoring the dynamics in between. However, recent advances in live cell single molecule imaging (SMI) (recently reviewed by Heinrich et al [15].) could provide new tools for mRNA export and quality control studies and further clarify the details of these processes.

Presence of factors with redundant functions, such as EJCs and RBPs in recruiting export receptors [20], and some others as cofactors and stabilizers such as Yra1/Aly [13,21,22] reinforces the possibility of the modulated-affinities mechanism hypothesis by implying that extra regulatory considerations are required for a successful quality control. Nonetheless, it is also conceivable to suggest that both mechanisms are in place to provide a reliable, efficient quality control. The possibility of presence of yet-to-be-identified (switch-like) proteins that signal the quality control proteins to inhibit the export of aberrant mRNAs are also not excluded [70].

One other aspect of mRNA quality control mechanism is whether it functions by selecting normal mRNAs (selection model), retaining aberrant mRNAs (retention model), or a combination of both [70]. In the case of the selection model, Mex67 and Mlp1 are found in complex and are suggested to indirectly interact with each other [38,57]. The selection model is also supported by observations that suggest the nuclear basket as an interaction platform for passing mRNPs [75]. Considering the wealth of information on the retention model, it is not conceivable to suggest that selection model is the sole mechanism for mRNA quality control. However, it could be the case that “retention” is the primary mechanism of quality control and “selection” further facilitates the process by providing a docking site for normal mRNAs to pass through the NPC more efficiently [54]. In line with the docking behavior hypothesis, it has been recently shown that SUN1, one of the components of the LINC complex (linker of the nucleus and the cytoplasm), has a significant role in mRNA export by interacting with the export receptors bound to mRNAs and eventually handing the mRNP to nuclear basket proteins for export [4,76].

Novel *in vivo* methods with higher spatial and temporal resolution, such as single particle RNA-imaging [15], are required to further refine these hypotheses (switch versus modulated affinities and selection versus retention) and identify the exact underlying molecular mechanisms. It is worth noting, however, that the mRNA quality control mechanism is still under investigation and the two hypotheses discussed here are not the only suggested mechanisms of mRNA quality control in the cell (for instance see [66]).
